# Angolensin Isolated from *Pterocarpus indicus* Willd. Attenuates LPS-Induced Sickness Behaviors in Mice and Exhibits CNS Safety

**DOI:** 10.3390/ijms26104887

**Published:** 2025-05-20

**Authors:** San Yoon Nwe, Peththa Wadu Dasuni Wasana, Hasriadi, Pasarapa Towiwat, Wisuwat Thongphichai, Boonchoo Sritularak, Suchada Sukrong

**Affiliations:** 1 Department of Pharmacognosy and Pharmaceutical Botany, Faculty of Pharmaceutical Sciences, Chulalongkorn University, Bangkok 10330, Thailand; sanyoonnwe@gmail.com (S.Y.N.); wisuwat.t@chula.ac.th (W.T.); boonchoo.sr@chula.ac.th (B.S.); 2 Center of Excellence in DNA Barcoding of Thai Medicinal Plants, Faculty of Pharmaceutical Sciences, Chulalongkorn University, Bangkok 10330, Thailand; 3 Department of Pharmacy, Faculty of Allied Health Sciences, University of Ruhuna, Galle 80000, Sri Lanka; dasuniwasana@ahs.ruh.ac.lk; 4 Animal Models of Chronic Inflammation-Associated Diseases for Drug Discovery Research Unit, Faculty of Pharmaceutical Sciences, Chulalongkorn University, Bangkok 10330, Thailand; adhiehasri@gmail.com (H.); pasarapa.c@chula.ac.th (P.T.); 5 Department of Pharmacology and Physiology, Faculty of Pharmaceutical Sciences, Chulalongkorn University, Bangkok 10330, Thailand; 6 Chulalongkorn School of Integrated Innovation, Chulalongkorn University, Bangkok 10330, Thailand

**Keywords:** *Pterocarpus indicus*, angolensin, rotarod, inflammation, sickness behaviors, central nervous system safety

## Abstract

Folk medicine in Thailand has long made use of *Pterocarpus indicus* Willd. for treating inflammation-related disorders. However, scientific exploration of isolated compounds from *P. indicus* for improving inflammation-associated sickness conditions and their impact on central nervous system (CNS) safety remain unexplored. The present study initially screened the anti-neuroinflammatory effects of angolensin, a compound isolated from *P. indicus* heartwood in vitro. Following substantial findings, the efficacy of angolensin was further evaluated in a mouse model of lipopolysaccharide (LPS)-induced sickness behaviors, alongside an assessment of its CNS safety profiles. The anti-neuroinflammatory effects of angolensin were evaluated in LPS-induced BV-2 microglial cells. The effects of angolensin on sickness behaviors were examined in LPS-induced mice using the Laboratory Animal Behaviors Observation, Registration and Analysis System (LABORAS). Proinflammatory cytokine expression in plasma samples of mice was also determined. LABORAS and rotarod tests were conducted to investigate its impact on the CNS. In vitro assessment of the anti-inflammatory activity of angolensin on BV-2 microglial cells revealed a concentration-dependent reduction in the release of LPS-induced nitric oxide (NO) and proinflammatory cytokines (TNF-α and IL-6). At a concentration of 20 µM, angolensin showed comparable results to the positive control, 20 µM minocycline. In mice, angolensin significantly improved LPS-induced sickness behaviors, as indicated by improved home-cage behaviors. Consistent with the in vitro findings, angolensin attenuated the release of proinflammatory cytokines in the plasma of LPS-induced mice. Importantly, angolensin did not induce any adverse effects on locomotion, motor coordination, or general well-being, indicating a favorable CNS safety profile. Overall, these results highlight the anti-inflammatory potential of angolensin in mitigating sickness behaviors in mice, while demonstrating its CNS safety.

## 1. Introduction

Sickness behavior comprises adaptive behavioral changes observed in both ill animals and humans during infection. The immune system plays a pivotal role in this process, often engaging in bidirectional communication with the brain, thereby impacting behavior, and aiding the recovery. Consequently, inflammatory cytokines released in response to tissue damage, invading pathogens, and other irritants are implicated in the occurrence of sickness behaviors [1]. The administration of lipopolysaccharide (LPS) can induce sickness behavior in rodents by stimulating the innate immune system [2,3]. This leads to an exaggerated neuroinflammatory response and the release of inflammatory cytokines, including interleukin-1 (IL-1), interleukin-6 (IL-6), and tumor necrosis factor-alpha (TNF-α), which contribute to sickness conditions [4]. These behaviors include anorexia, loss of daily activities, fatigue, sleep disturbances, reduced physical activities, and hyperalgesia [5,6]. Among these, the sensation of pain, a major symptom in every medical condition, significantly diminishes the quality of life and is associated with various pathogenic mechanisms [7]. Presently, nonsteroidal anti-inflammatory drugs (NSAIDs) and steroids are commonly used to manage sickness behaviors. However, considering the suboptimal therapeutic profiles and side effects of current drugs used in the management of pain and inflammation associated with sickness, the discovery and development of new drugs from natural sources, with fewer side effects and greater effectiveness, have become areas of growing interest [8].

Some species within the genus *Pterocarpus*, belonging to the Fabaceae family, have long been used in traditional medicine for their anti-inflammatory and analgesic properties. For instance, a decoction of *P. erinaceus* has traditionally been used to alleviate toothaches [9] and headaches [10]. *P. indicus*, another species from this genus, has been traditionally used as an analgesic to relieve pain associated with various ailments, including headaches, muscle pain, and menstrual cramps [11]. The aqueous extract of *P. indicus* has demonstrated anti-inflammatory activity in rats with atopic dermatitis induced by 2,4-dinitrochlorobenzene [11]. Previous reports have indicated that the ethanolic extract of *P. indicus* shows a strong analgesic effect by increasing the pain threshold in animals [12]. Another study demonstrated the potential analgesic activity of the aqueous extract of *P. indicus* in mice with acetic acid-induced pain [13]. These in vivo studies on the activity of *P. indicus* extracts support its traditional uses. While these medicinal properties of *P. indicus* are attributed to various bioactive compounds, including flavonoids, tannins, and terpenoids, there is still a lack of in-depth studies on the effects of individual phytochemical constituents of *P. indicus* on pain and inflammation associated with sickness conditions.

Angolensin, a methyl deoxybenzoin, has been isolated from the heartwood of various *Pterocarpus* species, including *P. angolensis* [14], *P. indicus* [15], and *P. erinaceus* [16]. Previous studies suggest that angolensin may contribute to a lower incidence of breast and prostate cancer due to its binding and transactivation properties at estrogen receptors [17]. Moreover, angolensin has exhibited antifungal activity [18], antimicrobial activity against methicillin-resistant *Staphylococcus aureus* strains [19], and α-glucosidase inhibition activity [20].

As angolensin is primarily extracted from the heartwood of *P. indicus*, further investigations into its biological activities, particularly in addressing sickness-related disorders, is imperative to substantiate the traditional use of *P. indicus* as an herbal remedy. The present investigation was undertaken on the bases of the abovementioned facts. Given the critical role of proinflammatory cytokines in the brain in the pathophysiology of sickness behavior [21,22], initial attempts were made to evaluate the in vitro anti-neuroinflammatory effects of angolensin on LPS-induced BV-2 microglia cells. Substantial findings obtained from the in vitro assays prompted the evaluation of the therapeutic efficacy of angolensin in a mouse model of LPS-induced sickness behaviors. Furthermore, its impact on motor coordination and general well-being were assessed to ascertain its central nervous system (CNS) safety profiles.

## 2. Results

### 2.1. Cytotoxicity Profiles of Angolensin in BV-2 Microglial Cells

**Figure 1:**
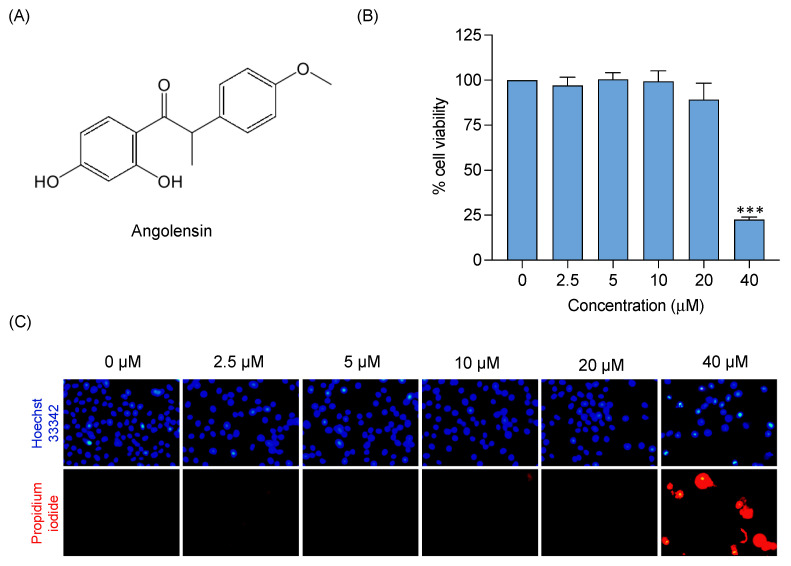
Chemical structure of angolensin (**A**), cytotoxicity profile of angolensin in BV-2 microglial cells, assessed by MTT assay (**B**), and apoptotic and necrotic characteristics of BV-2 microglial cells after treatment with angolensin, visualized using double staining assay (Hoechst 33342/Propidium Iodide) (×200 magnification) (**C**). *** *p* < 0.001 compared to the control group; one-way ANOVA followed by Dunnett’s post hoc test.

A cytotoxicity assessment of angolensin (Figure 1A) was performed to determine its non-toxic concentrations in BV-2 microglial cells. The cells were incubated with a series of angolensin concentrations (0, 2.5, 5, 10, 20, and 40 µM) for 24 h. The results indicated that 40 µM of angolensin significantly reduced cell viability compared to the control group (DMSO 0.5%), indicating cytotoxicity. Concentrations ≤ 20 µM showed no significant differences in the cell viability compared to the control group, suggesting no cytotoxic effects on the cells (Figure 1B). Further, the Hoechst 33342 and propidium iodide (PI) staining did not show apoptosis and necrosis in the cells treated with 0–20 µM angolensin (Figure 1C). Therefore, concentrations ≤ 20 µM were selected for further assessment of anti-neuroinflammatory activity.

### 2.2. In Vitro Anti-Inflammatory Effects of Angolensin in LPS-Stimulated BV-2 Cells

**Figure 2:**
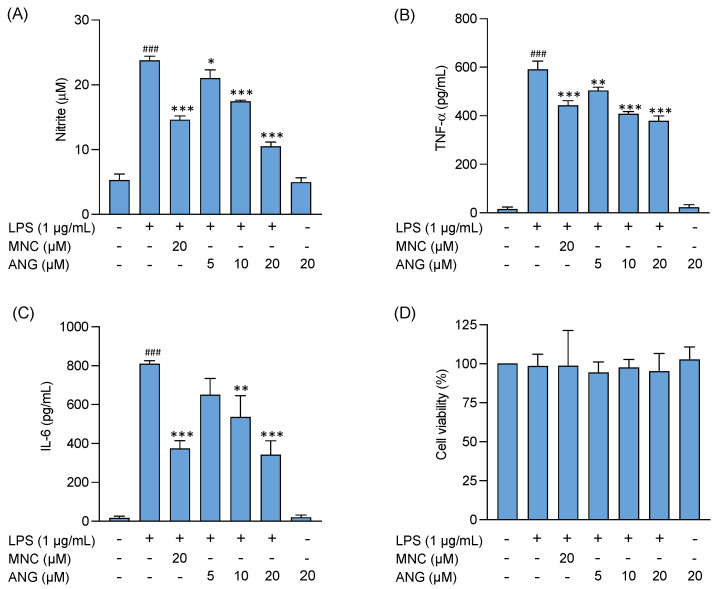
Effects of angolensin on the release of proinflammatory mediators in LPS-stimulated BV-2 microglial cells. Impact of the treatments on nitric oxide production (**A**), TNF-α levels (**B**), and IL-6 levels (**C**), and cell viability (**D**). Data are presented as mean ± SD (*n* = 3). ### *p* < 0.001 vehicle group vs. LPS group. * *p* < 0.05, ** *p* < 0.01 *** *p* < 0.001 compared to the LPS group; one-way ANOVA followed by Bonferroni post hoc test.

The effects of angolensin on the levels of proinflammatory mediators (NO, TNF-α, and IL-6) were evaluated in LPS-stimulated BV-2 microglial cells, an established in vitro model for assessing anti-neuroinflammatory activity. As shown in Figure 2A–C, LPS stimulation significantly increased the release of proinflammatory mediators (NO, TNF-α, and IL-6) compared to the control group. Treatment with angolensin demonstrated a concentration-dependent reduction in the release of proinflammatory mediators induced by LPS. Comparable results in suppressing proinflammatory mediators were observed between 20 µM minocycline and 20 µM angolensin.

Additionally, none of the treatments exhibited a significant effect on the cell viability (Figure 2D), indicating that the observed reductions in proinflammatory mediator levels in the LPS-stimulated BV-2 cells were not due to the decrease in cell viability or proliferation.

### 2.3. The Effect of Angolensin on LPS-Induced Sickness Behaviors

**Figure 3:**
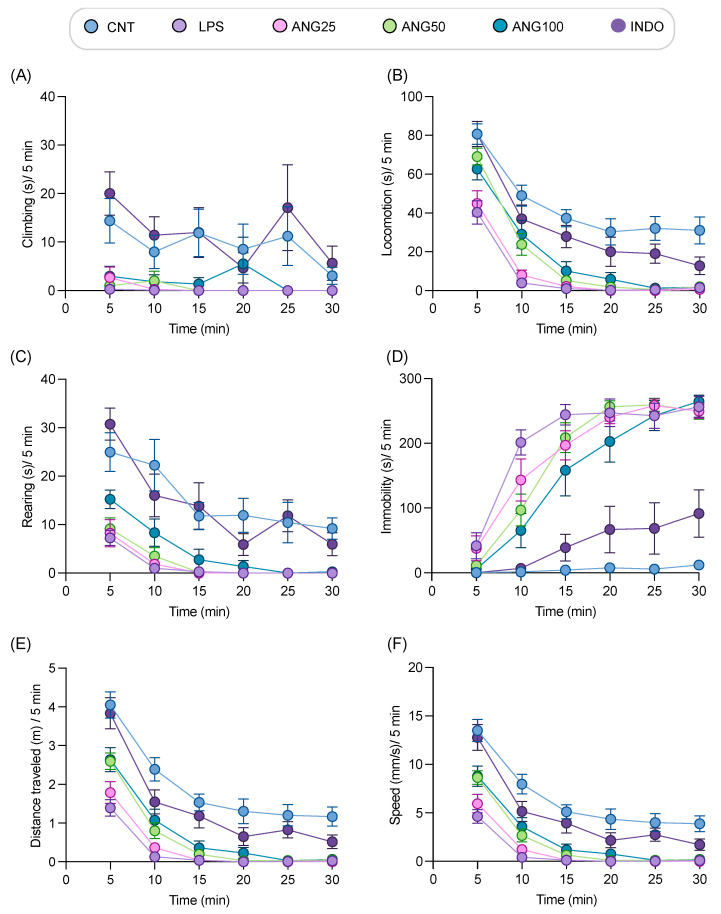
Time course effect of angolensin on exploratory behaviors in LPS-induced mice. The behaviors were presented as duration of climbing (**A**), locomotion (**B**), rearing (**C**) immobility (**D**), distance traveled (**E**), and speed (**F**). Data are expressed as mean ± SEM (eight mice per group). The differences between groups were analyzed by one-way ANOVA followed by the Bonferroni post hoc test. CNT (vehicle-treated mice, vehicle control), LPS (LPS-induced mice, disease control), ANG25 (angolensin 25 mg/kg), ANG50 (angolensin 50 mg/kg), ANG100 (angolensin 100 mg/kg), INDO (indomethacin 10 mg/kg).

Following promising results from the in vitro assays, LABORAS was used to investigate the effects of angolensin on the locomotor behaviors in LPS-induced mice (Figure 3 and Supplementary Figure S3). Analysis, conducted at 5-min intervals revealed a significant reduction in locomotive behaviors (Figure 3A–C) and a marked increase in immobility (Figure 3D) in LPS-treated mice compared to the control group. Additionally, the mice induced with LPS showed decreased distance traveled and reduced speed compared to the vehicle-treated mice (Figure 3E,F). After the treatment with different doses of angolensin (25, 50, and 100 mg/kg, i.p.), the mobile behaviors, speed, and distance traveled markedly improved compared to that of the LPS-induced mice. A dose-dependent reduction in immobility was also observed compared to the LPS-treated group (Figure 3A–F).

**Figure 4:**
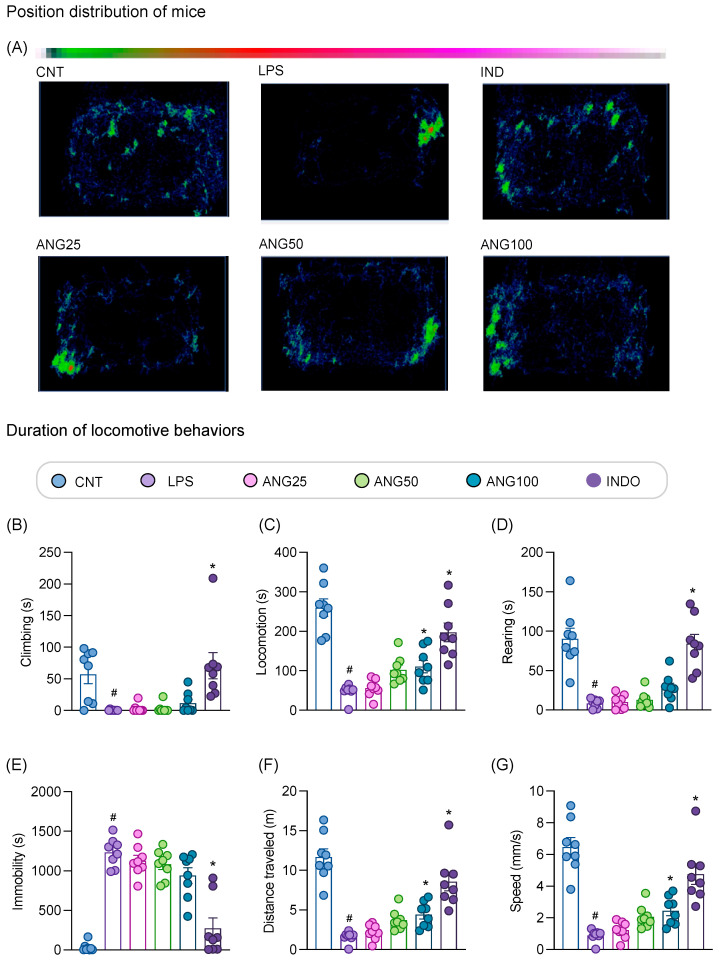
Effect of angolensin on exploratory behaviors in lipopolysaccharide (LPS)-induced mice. The position distribution of mice in the home-cage environment (**A**). Summary of behaviors presented as the duration of climbing (**B**), locomotion (**C**), rearing (**D**), immobility (**E**), and characteristics of locomotive behaviors presented as distance traveled (**F**) and speed (**G**). Data are expressed as mean ± SEM (eight mice per group). # *p* < 0.05, compared to the vehicle-treated group and * *p* < 0.05 compared to the LPS-induced group; one-way ANOVA followed by Bonferroni post hoc test. CNT (vehicle-treated mice, vehicle control), LPS (LPS-induced mice, disease control), ANG25 (angolensin 25 mg/kg), ANG50 (angolensin 50 mg/kg), ANG100 (angolensin 100 mg/kg), INDO (indomethacin 10 mg/kg).

The position distribution pattern of the mice in the home-cage environment and the cumulative effects of angolensin on exploratory behaviors over 30 min observation period are shown in Figure 4. According to the position distribution pattern, control mice explored the entire home-cage, whereas LPS-treated mice remained static in one spot and rarely explored the home-cage. Conversely, the mice treated with the positive control, indomethacin, actively explored the entire home-cage arena. Moreover, the LPS-induced mice treated with angolensin showed improved exploratory behaviors in the home-cage environment in a dose-dependent manner (Figure 4A). Quantitative analysis of the locomotive behaviors over a 30 min period indicates a dose-dependent improvement in all locomotive behaviors. Notably, the locomotion, speed, and distance traveled showed significant improvements with the administration of angolensin (100 mg/kg) (Figure 4B–G).

### 2.4. The Effects of Angolensin on the Expression of Proinflammatory Cytokines in LPS-Induced Mice

**Figure 5:**
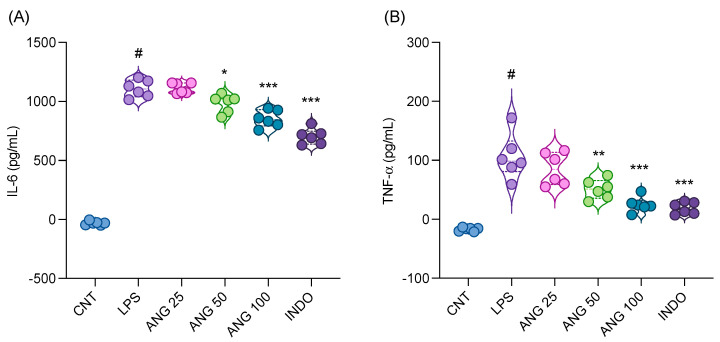
Biochemical analysis of proinflammatory cytokine levels in plasma. The effect of angolensin on LPS-induced IL-6 expression (**A**) and TNF-α expression (**B**) in plasma, as analyzed by ELISA. Data are expressed as mean ± SEM. # *p* < 0.001, compared to the vehicle-treated group and * *p* < 0.05, ** *p* < 0.01, and *** *p* < 0.001 compared to the LPS-induced group; one-way ANOVA followed by Bonferroni post hoc test. CNT (vehicle-treated mice, vehicle control), LPS (LPS-induced mice, disease control), ANG25 (angolensin 25 mg/kg), ANG50 (angolensin 50 mg/kg), ANG100 (angolensin 100 mg/kg), INDO (indomethacin 10 mg/kg).

The effect of angolensin on inflammatory pathways associated with sickness behavior was assessed by evaluating the expression levels of proinflammatory cytokines (IL-6 and TNF-α) in plasma samples. In the LPS-induced group, both IL-6 and TNF-α levels were significantly elevated compared to the control group. However, treatment with angolensin showed a marked, dose-dependent reduction in the levels of both IL-6 and TNF-α. Notably, the highest dose of angolensin (100 mg/kg) reduced TNF-α expression to a level comparable to that observed in the positive control group treated with 10 mg/kg indomethacin (Figure 5). The correlation analysis between cytokine expression and locomotive behaviors indicates a negative correlation between the plasma cytokine levels and the duration of climbing, locomotion, rearing, distance traveled, and speed. Conversely, there is a positive correlation with the duration of immobility. Notably, the plasma IL-6 expression shows a significant correlation with both the distance traveled and speed, while the plasma TNF-α expression significantly correlates with the duration of locomotion, immobility, distance traveled, and speed (Supplementary Table S1). These results suggest that observed improvements in locomotive behaviors in LPS-injected mice are likely mediated by angolensin’s ability to reduce plasma proinflammatory cytokine expression.

### 2.5. The Effects of Angolensin on General Behaviors and Well-Being of Mice

**Figure 6:**
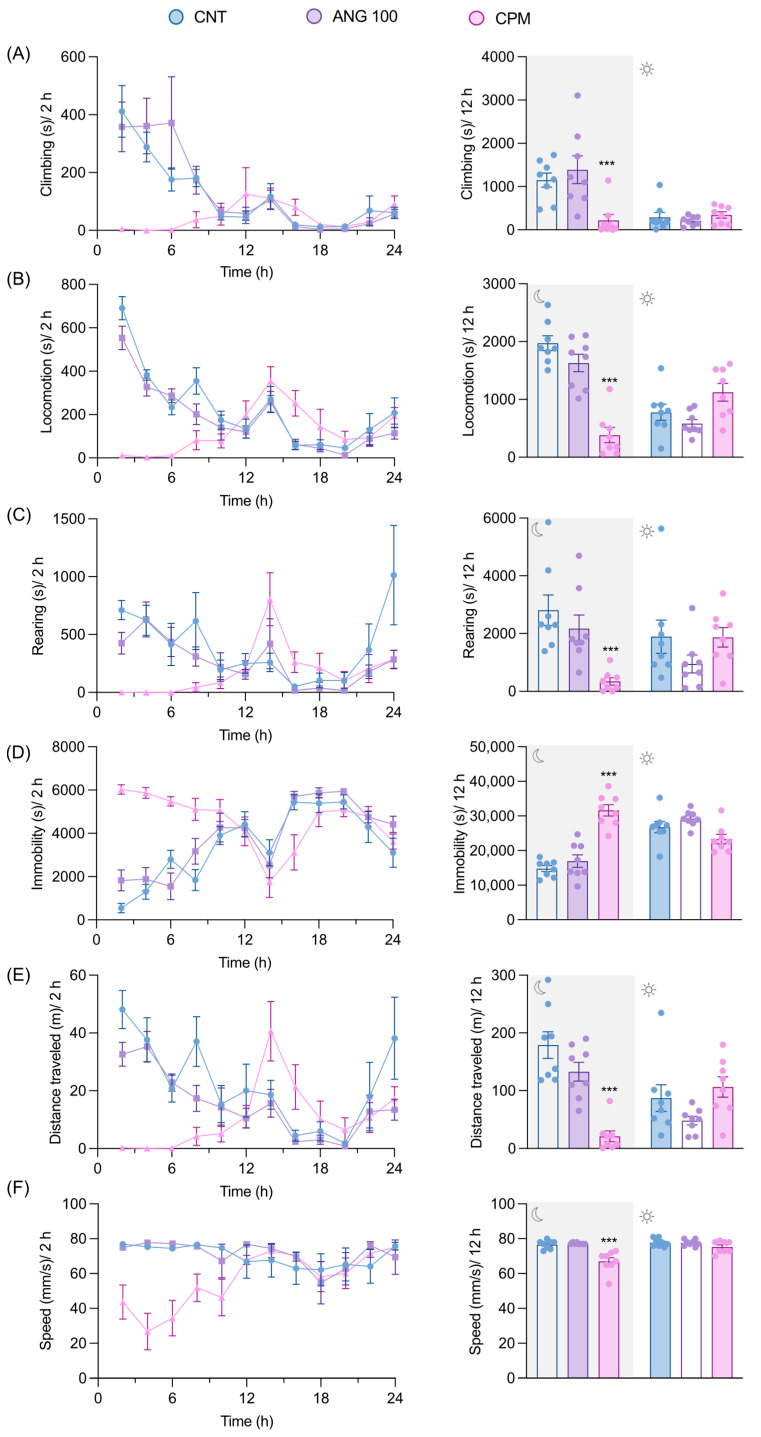
Effect of angolensin on the general behaviors of mice. Mice were administered the vehicle, angolensin (100 mg/kg), and chlorpromazine (5 mg/kg) intraperitoneally, and their home-cage behaviors were measured for 24 h. Time course effects of the treatments are shown at 2-h intervals, and summary data for nighttime and daytime (12-h intervals) are presented separately for each home-cage behavior: climbing (**A**), locomotion (**B**), rearing (**C**), immobility (**D**), distance traveled (**E**), and speed (**F**). Data are presented as mean ± SEM (*n* = 8 mice per group). *** *p* < 0.001 compared to the vehicle-treated group; one-way ANOVA followed by Dunnett’s post hoc test. CNT (vehicle-treated mice), ANG100 (angolensin 100 mg/kg), CPM (chlorpromazine 5 mg/kg).

**Figure 7:**
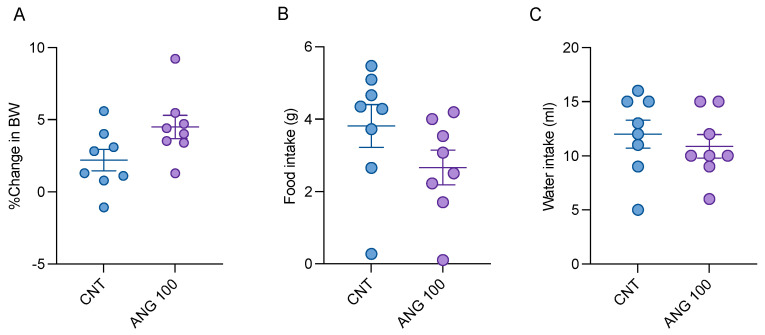
The effect of angolensin on the general well-being of mice is indicated as effect on body weight (**A**), food intake (**B**), and water intake (**C**). Data are presented as mean ± SEM (*n* = 8 mice per group). No significant differences were observed between the angolensin-treated group and the vehicle-treated group for any of the parameters tested (Student’s *t*-test). CNT, 5% DMSO; ANG100, angolensin (100 mg/kg).

LABORAS, an automated system for home-cage behavioral monitoring, was employed to evaluate the general behavior and well-being of mice. Long-term locomotion during nighttime, changes in body weight, and food and water intake were evaluated. Since mice are nocturnal animals, they exhibited more activity in their home-cage at night than during the day. Animals treated with the CNS depressant chlorpromazine displayed significantly more immobility and less locomotive behaviors during the night when compared to the vehicle-treated mice. However, there were no significant differences in the mobile behaviors observed during the daytime. Additionally, there were no significant differences found in any behaviors, including climbing, locomotion, rearing, immobility, average speed, and distance traveled, between the angolensin-treated group and the vehicle-treated group (Figure 6). The records of food and water intake as well as changes in body weight also indicated no significant differences between the angolensin and control groups (Figure 7).

### 2.6. The Effect of Angolensin on Motor Coordination

**Figure 8:**
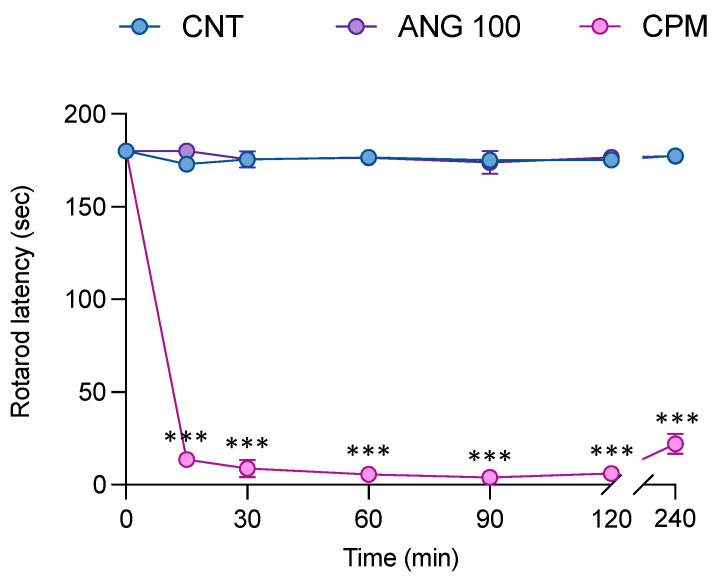
The effect of angolensin on motor coordination in mice. Mice were treated with vehicle, angolensin (100 mg/kg), and chlorpromazine (5 mg/kg, CPM) intraperitoneally, and rotarod latency was measured at 15, 30, 60, 90, 120, and 240 min post-treatment. The data are displayed as mean ± SEM (*n* = 8 mice per group). *** *p* < 0.001 compared to the vehicle-treated mice; one-way ANOVA followed by Dunnett’s post hoc test.

Administration of angolensin at the highest dose did not affect the ability of the mice to coordinate their movements (Figure 8), suggesting that angolensin does not induce sedative effects. Conversely, the mice in the chlorpromazine-administered group showed a significant decrease in rotarod latency 15 min after the treatment, which persisted until 240 min.

## 3. Discussion

Angolensin, a natural deoxybenzoin compound mainly isolated from *P. indicus*, has rarely been studied for its biological activities, except for its antifungal activities [23] and selective binding affinity to ERα/β heterodimers [24]. This study revealed that angolensin reduced the levels of nitric oxide and proinflammatory cytokines (TNF-α and IL-6) in LPS-induced BV-2 cells. Microglial BV-2 cells are part of the innate immune system within the healthy CNS [25]. The bacteria endotoxin LPS is commonly used experimentally to activate microglial cells. These activated microglial cells release proinflammatory cytokines that may contribute to pain and behavioral changes associated with sickness [26].

LPS has also been frequently used in experimental studies to induce fever and sickness [27]. Its administration significantly induces the release of proinflammatory mediators including chemokines, cyclooxygenase-2, cytokines, and nitric oxide by activating toll-like receptors (TLR-4) [28]. The intraperitoneal injection of LPS induces the expression of the cytokines IL-1β, IL-6, and TNF-α, which serve as primary mediators of sickness behaviors including pain in both peripheral and central nervous systems [29]. Comparison between sickness behaviors and mood changes in animals and humans following LPS administration have revealed substantial similarities [5]. Due to the induction of sickness behaviors and hyperalgesia, the use of LPS has been validated as a model for evaluating potential analgesic candidates in clinical trials [7]. In line with this, the LPS-induced mouse model is used in this study to evaluate the efficacy of angolensin.

In recent studies, LABORAS has been used as an alternative monitoring method to measure long-term locomotor activity and non-reflexive pain, serving as an indirect assessment of pain. The use of LABORAS for evaluating sickness behaviors in LPS-induced mice has been previously validated. In our previous studies, LPS-induced mice exhibited impaired locomotive behaviors compared to control mice [30]. The model was further validated by using indomethacin as a positive control to investigate innate non-reflexive pain-related behavioral changes in inflammatory mouse models. Administration of indomethacin notably improved locomotive behaviors in LPS-induced mice [30,31]. Therefore, in this study, the effects of angolensin on LPS-induced sickness behaviors in mice were observed using LABORAS, with indomethacin serving as the positive control. Male mice were selected to avoid confounding factors that might affect the results. A known limitation of the LABORAS is the individual placement of mice in cages, which can induce stress due to social isolation. This stress can influence analgesia/hyperalgesia and affect the interpretation of sickness behaviors. Male mice were chosen over female mice because females are more susceptible to stress from social isolation [32,33], which can lead to significant physiological changes [34,35], and because their locomotor activity varies with the estrus cycle [36]. However, it will be beneficial to evaluate how the gender of mice affects the therapeutic efficacy of angolensin in future studies. The findings indicated that angolensin improved sickness behaviors in mice, as indicated by improved mobile behaviors and reduced immobility in a dose-dependent manner. Furthermore, persistent infections and ongoing peripheral inflammation can lead to the development of clinical depression on top of sickness behavior [37]. Consequently, future research should investigate the impact of angolensin on depression and anxiety behaviors to fully understand its diverse therapeutic potential.

In many CNS disorders, such as Alzheimer’s disease, Parkinson’s disease, and multiple sclerosis, the disruption of the blood–brain barrier (BBB) facilitates the entry of peripheral immune mediators and therapeutic agents into the brain [38]. This pathological alteration may enhance the therapeutic efficacy of compounds that are otherwise poorly BBB-permeable under normal physiological conditions. Similarly, systemic administration of LPS can induce BBB disruption; however, the extent of this effect depends on the dosage used. Banks et al. (2015) demonstrated that only a high dose of LPS (3 mg/kg), but not lower doses such as 0.3 or 0.03 mg/kg, resulted in significant BBB disruption [39]. These findings suggest that the BBB is relatively resistant to LPS-induced permeability changes and that substantial disruption requires a relatively high LPS dose. In our study, a lower dose of LPS (200 µg/kg) was used to induce sickness behavior, consistent with prior research [40]. At this dose, it is likely that LPS activates neuroimmune pathways and elicits behavioral and inflammatory responses without causing significant BBB disruption. Therefore, the effects of angolensin observed in this study are more likely attributed to its modulation of peripheral and central inflammatory signaling rather than enhanced CNS access due to BBB breakdown. Nonetheless, future studies employing direct BBB permeability assays and models with intact BBBs will be essential to further evaluate the CNS pharmacokinetics and therapeutic potential of angolensin.

The cellular mechanisms underlying sickness behaviors are progressively being identified. The onset of sickness occurs with the release of proinflammatory cytokines by peripheral phagocytic cells upon encountering invading microorganisms. These immune signals from the periphery communicate with the brain through both a rapid neural pathway and a slower humoral pathway. Consequently, macrophage-like cells and microglia within the brain also express proinflammatory cytokines [2,41]. Therefore, this study aimed to evaluate the effect of angolensin on LPS-induced proinflammatory cytokine production to better understand its underlying mechanism in alleviating sickness behaviors. As anticipated, LPS significantly increased the expression of inflammatory cytokines in both BV-2 cells and plasma samples of mice. However, angolensin notably inhibited the release of these mediators (TNF-α and IL-6) both in vitro and in vivo. The observed inhibitory effect of angolensin on the release of inflammatory mediators may be the root cause of the improvements seen in sickness behaviors in LPS-induced mice. This finding is particularly significant as the release of proinflammatory cytokines by activated peripheral and central immune cells constitutes a major aspect of the pathophysiology of sickness behaviors.

The evaluation of newly developed compounds for their effects on the CNS is crucial to safeguard against potential harmful effects, as the CNS stands as one of the most vital organ systems [42]. Adverse effects commonly associated with NSAIDs, including vertigo, dizziness, recurrent falls, disorientation, headache, and encephalopathy, exhibit a significant correlation with CNS dysfunction [43]. Similarly, the majority of the adverse effects of opioid analgesics, such as sedation, nausea, vomiting, and mood alterations are attributed to their influence on the CNS [44]. Pharmacokinetic predictions using the SwissADME database suggest that angolensin is capable of crossing the BBB and is unlikely to be a substrate of P-glycoprotein (Supplementary Table S2), indicating its potential to exert CNS-related effects. Based on this rationale, CNS safety pharmacological assessments of angolensin were conducted in this study.

Studies have shown that the locomotive behaviors of mice hold clinical relevance to understanding adverse effects within the human CNS. For instance, disruptions in locomotor activity and rearing in mice have been linked to the experience of dizziness in humans. Similarly, impaired home-cage behaviors in rodents serve as a model for simulating somnolence and fatigue in humans [45]. In line with these findings, the application of LABORAS for assessing CNS safety in pharmacology has been previously validated and introduced [46]. Moreover, postural balance stands out as a crucial function of the CNS [47], prompting investigations into the correlation between adverse CNS effects and impaired motor coordination [48,49]. Accordingly, the rotarod test was assessed to evaluate the impact of the test compound on motor coordination. For the CNS safety evaluation of angolensin, locomotive behaviors and food and water intake of mice were investigated using LABORAS. The administration of the highest dose of angolensin used in the LPS-induced model did not exert a significant impact on spontaneous locomotor activity, general well-being evaluated by LABORAS, or rotarod latency, evaluated by the rotarod test in comparison to the control groups. Overall, the results suggest that angolensin could improve LPS-induced sickness behaviors in mice while demonstrating a favorable CNS safety profile. However, to obtain a more comprehensive understanding of its CNS safety, further pharmacological investigations are recommended in accordance with the European Medicines Agency (EMA) guidelines outlined in ICH S7A [50], including histopathological evaluations of brain tissue. Additionally, studies on blood–brain barrier permeability are warranted to assess the potential of angolensin for therapeutic use in other CNS-related disorders.

## 4. Materials and Methods

### 4.1. Sample Collection and Authentication

The plants were collected from the Faculty of Pharmaceutical Sciences, Chulalongkorn University, and authenticated by Associate Professor Boonchoo Sritularak (Ph.D) through comparison with the database of the Botanical Garden Organization. A voucher specimen (SS-1105) has been stored at the Museum of Natural Medicines, Faculty of Pharmaceutical Sciences, Chulalongkorn University, Bangkok, Thailand.

### 4.2. Isolation of Angolensin

The dried heartwood of *P. indicus* was finely powdered and macerated with ethyl acetate (EtoAc) at room temperature until the plant material was exhausted. The resulting extract was concentrated under reduced pressure using a rotatory evaporator at 45 °C to yield the EtoAc extract. This extract was then subjected to fractionation on a silica gel column (10 × 22 cm) using a gradient mixture of *n*-hexane–EtoAc (ranging from 1:0 to 0:1) followed by EtoAc:MeOH (ranging from 20:1 to 1:1). Five fractions (A–E) were collected for further separation. Fraction B was separated on a silica gel column using *n*-hexane–acetone (9:1) as the eluent, resulting in two subfractions (B1–B2). Subfraction B2 was further fractionated by column chromatography (silica gel, MeOH:CH_2_CL_2_ gradient), producing five more fractions (B21–B25). Fraction B24 was then purified on a Sephadex LH-20 column using MeOH as the eluent, ultimately yielding angolensin (Supplementary Figure S4). The identity of angolensin was confirmed by a comparing its NMR spectra (Supplementary Figures S1 and S2, and Supplementary Table S3) with previously published data [51].

### 4.3. Cell Culture

BV-2 microglia cells were purchased from AcceGen Biotechnology (North Brunswick, NJ, USA) and cultured in Dulbecco’s modified Eagle’s medium (DMEM) supplemented with 10% fetal bovine serum, 100 U/mL penicillin, and 100 mg/mL streptomycin. The cells were maintained at 37 °C in a humidified incubator with 5% CO_2_.

### 4.4. MTT Assay

The assessment of cell viability was performed using the 3-(4,5-dimethylthiazol-2-yl) 2,5-diphenyltetrazolium bromide (MTT) assay (Sigma-Aldrich, St. Louis, MO, USA). BV-2 cells were treated with different concentrations of the compounds for 24 h, followed by incubation with MTT solution (0.5 mg/mL) for 3 h. The resulting formazan crystals were dissolved in dimethyl sulfoxide (DMSO), and absorbance was measured at 540 nm using a CLARIOstar^®^ microplate reader (BMG Labtech, Ortenberg, Germany).

### 4.5. Double Staining Assay

A dual-fluorescence staining protocol using Hoechst 33,342 and Propidium Iodide (PI) (Sigma-Aldrich, St. Louis, MO, USA) was executed to distinguish apoptotic and necrotic characteristics in the investigated cells. Hoechst dye stains all cellular constituents, both viable and dead, while PI selectively stains the dead cells. Apoptotic cells exhibit distinctive morphological features, such as condensed and fragmented nuclei, following Hoechst staining. In contrast, necrotic cells display red fluorescence. Cells were seeded in 24-well plates at a density of 200,000 cells per well and treated with various concentrations of angolensin (0, 2.5, 5, 10, 20, and 40 µM), followed by staining with Hoechst 33,342 and PI for 15 min. The stained cells were observed under a fluorescence microscope (Olympus IX51 inverted microscope, Tokyo, Japan).

### 4.6. Cell Treatment and LPS Stimulation

A cytotoxicity assay was conducted to establish non-toxic concentrations of angolensin in BV-2 cells. Initially, BV-2 cells were seeded in 96-well plates at a density of 2 × 10^4^ cells per well. These cells were then exposed to varying angolensin concentrations (0, 2.5, 5, 10, 20, and 40 µM) following 24 h of seeding. After an additional 24 h, cell viability was evaluated using the MTT assay. Non-toxic concentrations were used to evaluate the efficacy of the compounds in BV-2 cells stimulated by LPS. BV-2 cells were pretreated with the non-toxic concentrations of angolensin for 1 h, followed by co-incubation with LPS (1 µg/mL) [40]. After 24 h, the culture medium was collected for determining cytokine levels (TNF-α and IL-6) and nitrite levels.

### 4.7. Nitrite Assay

The nitrite assay was performed to investigate the effect of angolensin on the nitric oxide (NO) level according to standard protocols provided by the manufacturers. Briefly, the media from treated cells were mixed with an equal amount of the Griess reagent in 96-well plates and incubated for 20 min at room temperature. The absorbance was measured using a microplate reader at a wavelength of 520 nm. NaNO_2_ was used to construct a standard curve to determine the nitrite levels in the media.

### 4.8. Cytokine Assay

The levels of cytokines released into the media (TNF-α and IL-6) following the indicated treatments were measured using enzyme-linked immunosorbent assay (ELISA) kits. The assays were conducted according to the instructions provided by the manufacturer (BioLegend, San Diego, CA, USA).

### 4.9. Animals

Male ICR mice (5–6 weeks old) were procured from Nomura Siam International Co., Ltd. (Bangkok, Thailand) and housed in the animal facility at the Faculty of Pharmaceutical Sciences, Chulalongkorn University. The mice were acclimatized for 1–2 weeks prior to experimentation to ensure proper adjustment to the environment. Animals were housed in groups of four to five mice per cage under standard laboratory conditions: a 12:12 h light/dark cycle, a temperature of 22 ± 2 °C, a humidity of 40–60%, and unlimited access to standard laboratory food and water. All protocols and procedures were approved by the Institutional Animal Care and Use Committee of the Faculty of Pharmaceutical Sciences, Chulalongkorn University (Protocol No. 22-33-025).

### 4.10. Drugs and Treatment Administration

**Figure 9:**
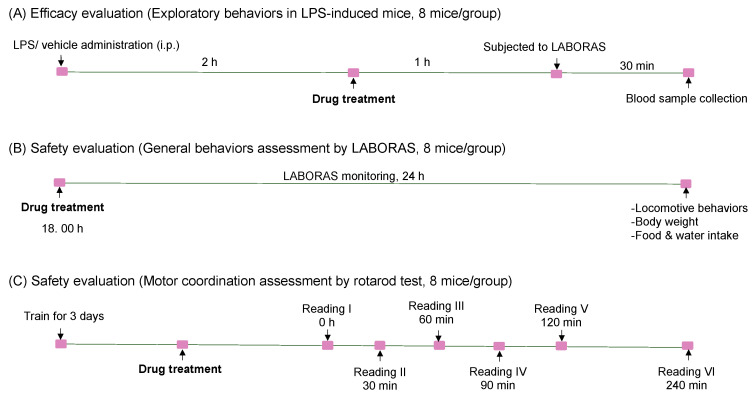
Schematic illustration of the experimental design and groups. (**A**) Mice subjected to LABORS automated home-cage monitoring to assess the therapeutic efficacy of angolensin against LPS-induced sickness behaviors. (**B**) Safety pharmacology evaluation of angolensin by assessing its effects on the general behaviors of mice. (**C**) Safety pharmacology evaluation of angolensin by assessing its effects on motor coordination in mice.

Angolensin was initially dissolved in DMSO (Sigma-Aldrich, St. Louis, MO, USA) and then diluted with normal saline to achieve a final DMSO concentration of 5%. The intraperitoneal route was used to administer all treatments, including angolensin at doses of 25, 50, and 100 mg/kg, and the vehicle (5% DMSO in normal saline), maintaining a constant volume of 10 mL/kg body weight. Indomethacin (10 mg/kg) was used as the positive control for the mouse model of LPS-induced sickness behaviors, while chlorpromazine (5 mg/kg) was used as the positive control for CNS safety evaluations. Both indomethacin and chlorpromazine were dissolved in normal saline and administered intraperitoneally. For each behavioral evaluation, 8 mice per group were used (Figure 9).

### 4.11. Assessment of Exploratory Behavior by LABORAS Automated Home-Cage Behavioral Analysis

The efficacy of angolensin in attenuating LPS-induced sickness behaviors in mice was evaluated, using the LABORAS automated behavioral analysis system, as previously reported in a study [30]. In this model, excessive immunological responses triggered by LPS injection were identified as the cause of reduced locomotor activity in mice. Before the test, mice underwent a one-hour acclimation period in the testing environment. Then, LPS (200 μg/kg) was administered intraperitoneally to induce sickness behavior [40]. After two hours, mice received intraperitoneal administrations of either vehicle, indomethacin (10 mg/kg), or various doses of angolensin (25 mg/kg, 50 mg/kg, and 100 mg/kg). An hour after treatment administration, each mouse was placed in the LABORAS automated home-cage to assess their locomotor activity for 30 min. The effects of the compounds on LPS-induced locomotor impairments were evaluated separately for locomotion (walking and running), climbing, rearing, immobility, speed, and distance traveled. The time spent on each behavior and the frequency of each behavior were compared.

### 4.12. Blood Sample Collection

Immediately after LABORAS behavioral evaluation, blood samples were collected from mice via cardiac puncture under isoflurane anesthesia. The procedure adhered to the guidelines of the Virginia Tech Institutional Animal Care and Use Committe (SOP: Blood Collection in the Mouse, Intracardiac, https://ouv.vt.edu/), accessed on 15 July 2023. Blood samples were centrifuged at 3000 rpm for 10 min at 4 °C to isolate plasma. The collected plasma was stored at −80 °C until use. Expression levels of cytokines (TNF-α and IL-6) in the plasma were measured using commercial ELISA kits (BioLegend, San Diego, CA, USA) according to the manufacturer’s instructions.

### 4.13. Assessment of General Well-Being of Mice by LABORAS Automated Home-Cage Monitoring

The effect of the highest dose of angolensin (100 mg/kg) on the general well-being of mice was evaluated using LABORAS automated home-cage long-term monitoring. LABORAS home-cages were equipped with predetermined amounts of food pellets and water. Following the administration of test compounds, mice were placed in the LABORAS home-cages immediately, and their spontaneous locomotive behaviors were recorded for 24 h. The test commenced at 18:00, considering the nocturnal characteristics of mice. After the locomotive behavioral measures, mice were removed from the cages, and data on body weight, remaining water volume, and remaining weight of the food were recorded.

### 4.14. Rotarod Test

The effect of the highest dose of angolensin (100 mg/kg) on motor coordination was assessed using the rotarod test. Animals were initially trained to stay on a rotating horizontal rod (17 rpm), and only those that successfully remained on the rotating rod for at least 180 s in three consecutive trials were included in the study. Following the administration of test compounds, mice were placed on the rotating rod at 30, 60, 90, 120, and 240 min post-compound administration. The latency to fall was recorded at each time point and expressed as the time (in seconds) that the mice remained on the rotarod. All experiments were carried out during the daytime (8.00–14.00).

### 4.15. Statistical Analysis

The results of all behavioral tests were presented as means ± SEM. Statistical analyses were performed using GraphPad Prism Version 10.2.2. One-way analysis of variance (ANOVA) followed by the Bonferroni or Dunnett’s post hoc test was used for multiple comparisons and Student’s *t*-test was employed for two group comparisons. Pearson’s correlation analysis was performed to examine the correlation between LPS-induced plasma cytokine expression and locomotive behavioral measures in mice. Statistical significance was considered when *p* < 0.05.

## 5. Conclusions

In summary, the study demonstrated that angolensin exhibits anti-neuroinflammation properties by reducing the expression of proinflammatory cytokines both in LPS-induced BV-2 microglial cells and in plasma samples of LPS-induced mice. These findings suggest that angolensin has the potential to attenuate both peripheral and central inflammatory responses. Moreover, angolensin did not induce adverse effects on motor coordination, spontaneous locomotor activity, or general well-being, suggesting a favorable CNS safety profile. However, further investigations are still needed to comprehensively understand the underlying mechanisms contributing to the therapeutic efficacy of angolensin in combating sickness behaviors. Furthermore, safety evaluations should be extended to other physiological systems, such as the respiratory and cardiovascular systems. It is also recommended to study the pharmacokinetics of angolensin after oral administration. These additional assessments, combined with our findings, will deepen our understanding of angolensin’s therapeutic potential, pharmacokinetics, and safety, paving the way for its clinical application. 

## Data Availability

This article and Supplementary Materials include the data used to support the findings of this study.

## Supplementary Materials

The following supporting information can be downloaded at https://www.mdpi.com/article/10.3390/ijms26104887/s1. References [51,52] are cited in the Supplementary Material.

## Abbreviations

LPS	Lipopolysaccharide
LABORAS	Laboratory Animal Behavior Observation, Registration and Analysis System
CNS	Central nervous system
TNF-α	Tumor necrosis factor-alpha
IL-6	Interleukin-6
NO	Nitric oxide
