# Supplementary material for: Angolensin Isolated from Pterocarpus indicus Willd. Attenuates LPS-Induced Sickness Behaviors in Mice and Exhibits CNS Safety
Source: Int J Mol Sci. 2025 May 20;26(10):4887. doi: 10.3390/ijms26104887 (PMC12111846; doi:10.3390/ijms26104887)
Supplement: Supplementary file 1 [file ijms-26-04887-s001.zip › ijms-3591090-supplementary.pdf]

Supplementary materials

Angolensin isolated from *Pterocarpus indicus* Willd. attenuates LPS-induced sickness behaviors in mice and offers CNS safety profiles

San Yoon Nwe [1,2], Peththa Wadu Dasuni Wasana [3], Hasriadi [4,5], Pasarapa Towiwat [4,5], Wisuwat Thongphichai [1,2], Boonchoo Sritularak [1], Suchada Sukrong [1,2,6] *

[1] Department of Pharmacognosy and Pharmaceutical Botany, Faculty of Pharmaceutical Sciences, Chulalongkorn University, Bangkok 10330, Thailand
[2] Center of Excellence in DNA Barcoding of Thai Medicinal Plants, Faculty of Pharmaceutical Sciences, Chulalongkorn University, Bangkok 10330, Thailand
[3] Department of Pharmacy, Faculty of Allied Health Sciences, University of Ruhuna, Galle 80000, Sri Lanka
[4] Animal Models of Chronic Inflammation-associated Diseases for Drug Discovery Research Unit, Faculty of Pharmaceutical Sciences, Chulalongkorn University, Bangkok 10330, Thailand
[5] Department of Pharmacology and Physiology, Faculty of Pharmaceutical Sciences, Chulalongkorn University, Bangkok 10330, Thailand
[6] Chulalongkorn School of Integrated Innovation, Chulalongkorn University, Bangkok 10330, Thailand

* Corresponding author: Suchada.su@chula.ac.th; Tel.: +6681-8196742

Supplementary Table S1. Pearson Correlation Between Cytokine Expression and Therapeutic Efficacy of Angolensin in LPS-Induced Sickness Behaviors

**Cytokine**	**Behavioral parameter**	**Corelation coefficient (r)**	**R squared**	**P value (two-tailed)**	**Significance**
**IL-6 (pg/mL)**	Climbing (s)	-0.9049	0.8189	0.0951	ns
	Locomotion (s)	-0.9355	0.8751	0.0645	ns
	Rearing (s)	-0.9449	0.8927	0.0551	ns
	Immobility (s)	0.9218	0.8497	0.0782	ns
	Distance travelled (m)	-0.9537	0.9096	0.0463	*
	Speed (mm/s)	-0.9537	0.9095	0.0463	*
**TNF-α (pg/mL)**	Climbing (s)	-0.8778	0.7705	0.1222	ns
	Locomotion (s)	-0.9718	0.9443	0.0282	*
	Rearing (s)	-0.8871	0.7869	0.1129	ns
	Immobility (s)	0.9678	0.9366	0.0322	*
	Distance travelled (m)	-0.9955	0.9911	0.0045	**
	Speed (mm/s)	-0.9955	0.9910	0.0045	**

ns, not significant; *$p < 0.05$, **$p < 0.001$ indicate significant correlation between cytokine expression and behavioral measures.

Supplementary Table S2. Predicted pharmacokinetic and drug interaction properties of angolensin based on *in silico* analysis using the SwissADME web tool (http://www.swissadme.ch/, accessed on 5 May 2025) [1].

**Pharmacokinetic/ Interaction Parameter**	**Prediction**
GI absorption	High
BBB permeant	Yes
Pgp substrate	No
CYP1A2 inhibitor	Yes
CYP2C19 inhibitor	Yes
CYP2C9 inhibitor	No
CYP2D6 inhibitor	No
CYP3A4 inhibitor	Yes

Supplementary Table S3. Comparison of ^{13}C and ^{1}H NMR of the reported data of angolensin [2] (200 MHz, in $CDCl_3$) and isolated angolensin (400 MHz, in $CDCl_3$).

**Position**	**Angolensin (200 MHZ, in CDCl3)**		**Angolensin in (400 MHz, in CDCl3)**	
	**δ**C (mult., *J* in Hz)	**δ**H (mult., *J* in Hz)	**δ**C (mult., *J* in Hz)	**δ**H (mult., *J* in Hz)
1	205.4		205.01	
2	45.9	4.57 (q, *J*=6.8 Hz)	45.9	4.57 (q, J=6.8 Hz)
3	19.2	1.49 (d, *J*=7.00 Hz)	19.2	1.49 (d, J= 6.8 Hz)
1′	112.8		112.9	
2′	162.8		162.4	
3′	103.5	6.27 (d, *J*=2.5)	103.5	6.27 (d, J= 8.8 Hz)
4′	165.6		165.8	
5′	108.1	6.32 (d, *J*=8.8 Hz)	107.7	6.38 (d, J=8.8 Hz)
6′	132.8	7.68 (d, 8.8 Hz)	132.6	7.68 (d, J=8.8 Hz)
1″	133.5		133.5	
2″	128.6	7.20 (d, *J*=8.7 Hz)	128.5	7.20 (d, J=8.8 Hz)
3″	114.4	6.84 (d, *J*=8.6 Hz)	114.3	6.81 (d, J=8.8 Hz)
4″	158.4		158.5	
5″	114.4	6.84 (d, *J*=8.6 Hz)	114.3	6.81 (d, J=8.8 Hz)
6″	128.6	7.20 (d, *J*=8.7 Hz)	128.5	7.20 (d, J=8.8 Hz)
-OMe	55.3	3.75 (s)	55.2	3.78 (s)
OH		12.00		12.89

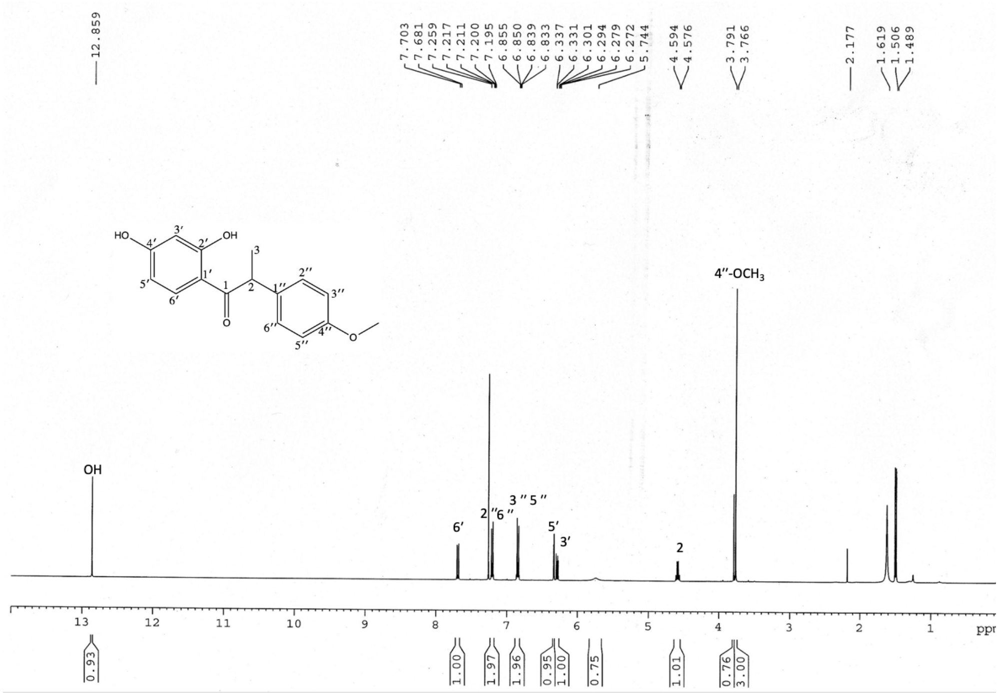

Supplementary Figure S1. ^{1}H-NMR (400 MHz, CDCL$_3$) spectrum of angolensin (ranging from 1.49-12.86).

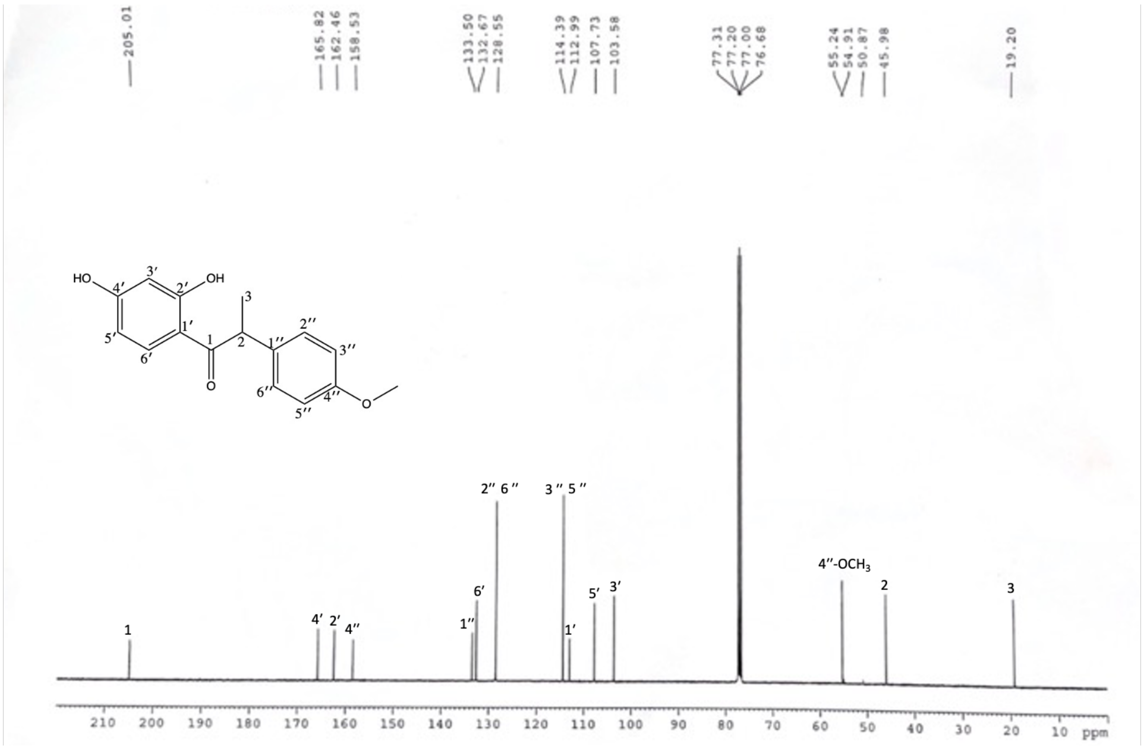

Supplementary Figure S2. ^{13}C-NMR (125 MHz, CDCL$_3$) spectrum of angolensin

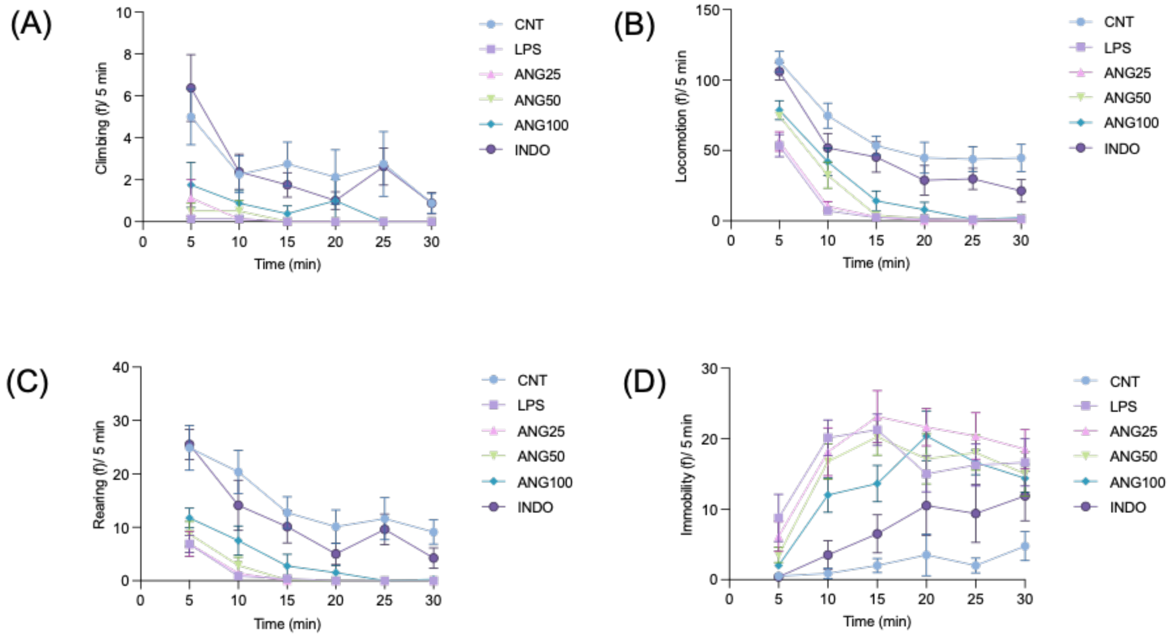

Supplementary Figure S3. Time course effect of angolensin on exploratory behaviors in LPS-induced mice. The behaviours were presented as frequency of climbing (A), locomotion (B), rearing (C) immobility (D). Data are expressed as mean ± SEM (eight mice per group). CNT (vehicle-treated mice, vehicle control), LPS (LPS-induced mice, disease control), ANG25 (angolensin 25 mg/kg), ANG50 (angolensin 50 mg/kg), ANG100 (angolensin 100 mg/kg), INDO (indomethacin 10 mg/kg).

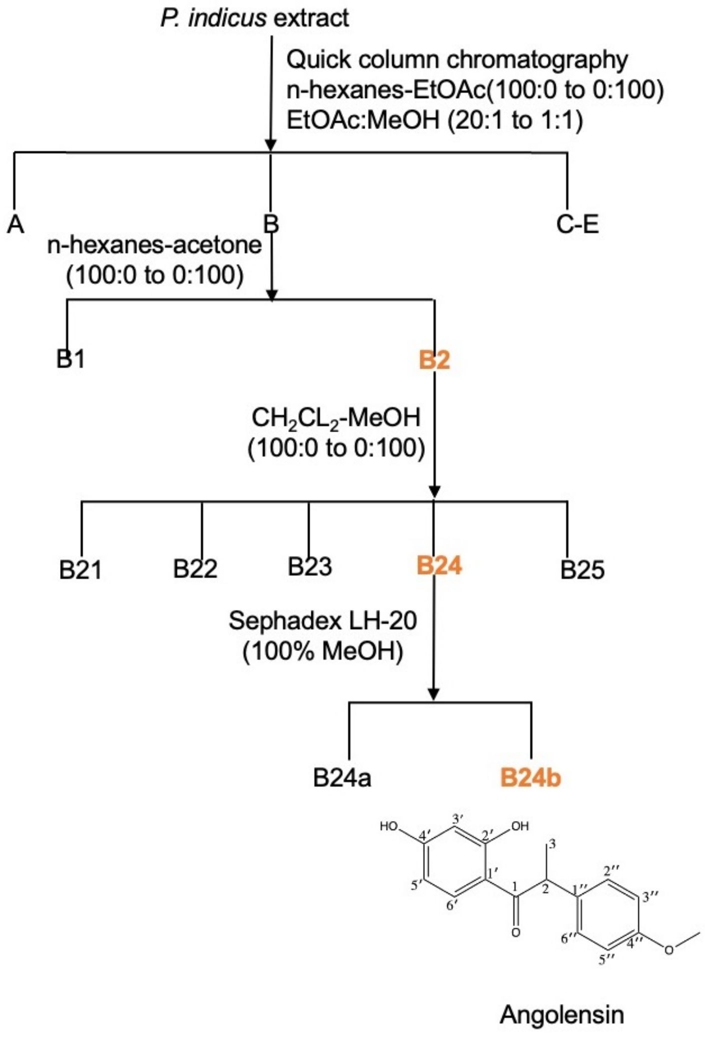

Supplementary Figure S4. Scheme for the isolation steps of angolensin from *P. indicus* heartwood.

Reference

[1] SwissADME: a free web tool to evaluate pharmacokinetics, drug-likeness and medicinal chemistry friendliness of small molecules. Sci. Rep. (2017) 7:42717.

[2] Salakka, A.; Wähälä, K. Synthesis of α-methyldeoxybenzoins. *J. Chem. Soc., Perkin trans*. 1 **1999**, (18), 2601-2604.
